# Serotonergic modulation of spatial working memory: predictions from a computational network model

**DOI:** 10.3389/fnint.2013.00071

**Published:** 2013-09-26

**Authors:** Maria Cano-Colino, Rita Almeida, Albert Compte

**Affiliations:** ^1^Systems Neuroscience Group, Institut d'Investigacions Biomèdiques August Pi i SunyerBarcelona, Spain; ^2^Department of Neuroscience, Karolinska InstituteStockholm, Sweden

**Keywords:** persistent activity, prefrontal cortex, computational model, metacognition, working memory

## Abstract

Serotonin (5-HT) receptors of types 1A and 2A are strongly expressed in prefrontal cortex (PFC) neurons, an area associated with cognitive function. Hence, 5-HT could be effective in modulating prefrontal-dependent cognitive functions, such as spatial working memory (SWM). However, a direct association between 5-HT and SWM has proved elusive in psycho-pharmacological studies. Recently, a computational network model of the PFC microcircuit was used to explore the relationship between 5-HT and SWM (Cano-Colino et al., 2013). This study found that both excessive and insufficient 5-HT levels lead to impaired SWM performance in the network, and it concluded that analyzing behavioral responses based on confidence reports could facilitate the experimental identification of SWM behavioral effects of 5-HT neuromodulation. Such analyses may have confounds based on our limited understanding of metacognitive processes. Here, we extend these results by deriving three additional predictions from the model that do not rely on confidence reports. Firstly, only excessive levels of 5-HT should result in SWM deficits that increase with delay duration. Secondly, excessive 5-HT baseline concentration makes the network vulnerable to distractors at distances that were robust to distraction in control conditions, while the network still ignores distractors efficiently for low 5-HT levels that impair SWM. Finally, 5-HT modulates neuronal memory fields in neurophysiological experiments: Neurons should be better tuned to the cued stimulus than to the behavioral report for excessive 5-HT levels, while the reverse should happen for low 5-HT concentrations. In all our simulations agonists of 5-HT_1A_ receptors and antagonists of 5-HT_2A_ receptors produced behavioral and physiological effects in line with global 5-HT level increases. Our model makes specific predictions to be tested experimentally and advance our understanding of the neural basis of SWM and its neuromodulation by 5-HT receptors.

## Introduction

Spatial working memory (SWM) is a function of the prefrontal cortex (PFC) (Smith and Jonides, 1999) that is known to be under the neuromodulatory control of the monoamine nuclei of the brainstem (Brozoski et al., 1979; Porrino and Goldman-Rakic, 1982; Ellis and Nathan, 2001; Robbins and Arnsten, 2009; Arnsten, 2011; Arnsten et al., 2012). Especially catecholamines have been implicated in SWM, while only comparatively fewer studies have investigated neuromodulation of SWM by the indolamine serotonin (5-hydroxytryptamine, 5-HT) (Park et al., 1994; Luciana et al., 1998, 2001; Vollenweider et al., 1998; Carter et al., 2005; Wingen et al., 2007; Wittmann et al., 2007; Mendelsohn et al., 2009; Silber and Schmitt, 2010). These studies have not found consistent effects of 5-HT on SWM, even if the serotonergic system is associated with general cognitive function (Schmitt et al., 2006; Robert and Benoit, 2008; Ögren et al., 2008; Froestl et al., 2012). This is unexpected, given the marked effect of local application of serotonergic drugs on the persistent activity of prefrontal neurons in monkeys engaged in a SWM task (Williams et al., 2002), which is believed to subserve working memory function in the PFC (Funahashi et al., 1989; Goldman-Rakic, 1995). A role for 5-HT in controlling PFC-dependent functions is also suggested by the strong expression of serotonin receptors in PFC (Jacobs and Azmitia, 1992; Jakab and Goldman-Rakic, 1998; Barnes and Sharp, 1999; Amargós-Bosch et al., 2004; Santana et al., 2004; De Almeida et al., 2008) and their capacity to modulate prefrontal cortical activity (Jacobs and Azmitia, 1992; Puig et al., 2004, 2005, 2010; Celada et al., 2013).

Recently, a computational model of SWM was used to study the effects of 5-HT receptors on network function (Cano-Colino et al., 2013). This study suggested that a non-monotonic dependency of SWM performance with 5-HT concentration could underlie the difficulty in identifying serotonergic effects in psychopharmacological studies of SWM. Furthermore, the network model predicted that worsened SWM performance upon excessive and defective activation of 5-HT receptors could be discriminated based on a careful examination of the nature of the errors committed (Cano-Colino et al., 2013). In particular, the confidence declared by subjects after erroneous responses could distinguish the behavioral effects of increased and reduced 5-HT activations, and thus lead to detecting a robust serotonergic effect on SWM. These predictions are experimentally testable, since confidence reports are being increasingly used in both human (Pessoa and Ungerleider, 2004; Mayer and Park, 2012; Rademaker et al., 2012) and animal (Middlebrooks and Sommer, 2011, 2012; Tanaka and Funahashi, 2012) studies of working memory. However, there are limitations in relying only on the confidence report to test the computational model. On the one hand, confidence reports rely on meta-cognition, the knowledge about one's own cognitive processes (Flavell, 1979), which is also a PFC-dependent function (Schnyer et al., 2004; Pannu et al., 2005; Rounis et al., 2010; Middlebrooks and Sommer, 2012). As a result, metacognitive reports could be themselves affected by serotonergic manipulations and there could be a confound between a 5-HT effect on the confidence circuits and a 5-HT effect on the SWM circuit. On the other hand, neurobiological research on metacognition is in its infancy, so that confidence-based predictions are difficult to test in animal studies.

Here, we present behavioral and physiological predictions from the computational model of SWM subject to 5-HT neuromodulation (Cano-Colino et al., 2013) that do not require metacognitive reports. In this way, we provide control predictions to disambiguate a 5-HT effect on metacognition as opposed to on SWM, and we also extend the predictive power of the computational model so it can be validated in electrophysiological studies that use well-established behavioral paradigms of SWM during application of pharmacological agents in behaving animals (Williams et al., 2002; Meneses, 2007; Vijayraghavan et al., 2007).

## Materials and methods

### The model network

We used a neuronal network model of the PFC to explore the relationship between 5-HT and SWM (Cano-Colino et al., 2013). The network model represents a local circuit of the monkey dorsolateral PFC (Funahashi et al., 1989). The local recurrent cortical network consists of two populations of leaky integrate-and-fire neurons (Tuckwell, 1988): excitatory pyramidal cells (*N*_*E*_ = 1024) and inhibitory interneurons (*N*_*I*_ = 256). The membrane voltage *V*_*m*_ of each neuron obeys the following dynamical equation:

$$C_{m}\frac{dV_{m}}{dt}=-I_{L}-I_{\text{syn},\;e}-I_{\text{syn},\;i}-I_{\text{ext}}+I_{s}-I_{\text{5-HT}}$$

where *C*_*m*_ represents the membrane capacitance of the neuron. When *V*_*m*_ reaches a threshold value *V*_*th*_, *V*_*m*_ is reset to *V*_res_ and stays there for an absolute refractory period τ_ref_. *I*_ext_ represents random synaptic inputs from outside the network, simulated as uncorrelated Poisson spike trains activating AMPA channels of conductance *g*_ext_ at a rate *n*_ext_. *I*_s_ is the input current associated with stimulus presentation (see below). *I*_syn, *e*_ and *I*_syn, *i*_ are the recurrent synaptic inputs from presynaptic pyramidal cells and interneurons, respectively. Details of synaptic transmission are given below. For pyramidal neurons *I*_5-HT_ is the current modulated by 5-HT (see below) and the leak current is *I*_*L*_ = *g*_*L*_ (*V*_*m*_ − *E*_*L*_), with *g*_*L*_ and *E*_*L*_ being the conductance and reversal potential of leak channels. For interneurons *I*_*L*_ depends on 5-HT concentration (see below) and there is no *I*_5-HT_. The intrinsic parameters that characterize pyramidal cells are: *C*_*m*_ = 0.5 nF, *g*_*L*_ = 27.4 nS, *E*_*L*_ = −70 mV, *V*_*th*_ = −50 mV, *V*_res_ = −60 mV, ν_ext_ = 1650 Hz, *g*_ext_ = 5 nS, and τ_ref_ = 2 ms. For interneurons *C*_*m*_ = 0.2 nF, *E*_*L*_ = −70 mV, *V*_*th*_ = −50 mV, *V*_res_ = −60 mV, ν_ext_ = 1800 Hz, *g*_ext_ = 1.8 nS, and τ_ref_ = 1 ms.

Neurons are connected through conductance-based synapses of the AMPA, NMDA, and GABA_A_ types, which were calibrated by the experimentally measured dynamics of synaptic currents (Wang, 1999). Thus, postsynaptic currents were modeled according to *I*_syn_ = *g*_syn_ *s*(*V*_*m*_ − *V*_syn_), where *g*_syn_ is a synaptic conductance, *s* a synaptic gating variable, and *V*_syn_ the synaptic reversal potential (*V*_syn_ = 0 for excitatory synapses, *V*_syn_ = −70 mV for inhibitory synapses). AMPAR and GABA_A_R synaptic gating variables were modeled as an instantaneous jump of magnitude of 1 when a spike occurred in the presynaptic neuron followed by an exponential decay with time constant 2 ms for AMPA and 10 ms for GABA_A_. The NMDA conductance was voltage-dependent, with *g*_syn_ multiplied by 1/(1 + [Mg^2+^] exp(−0.062 *V*_*m*_)/3.57), [Mg^2+^] = 1.0 mM, and the channel kinetics were modeled by:

$$\begin{array}{l} \frac{ds}{dt}=\frac{-1}{\tau_{s}}s+\alpha_{s}x\left(1-s\right)\text{​},\\ \frac{dx}{dt}=\frac{-1}{\tau_{x}}x+\sum_{i}\delta \left(t-t_{i}\right) \end{array}$$

where *s* is the gating variable, *x* a synaptic variable proportional to the neurotransmitter concentration in the synapse, *t*_*i*_ the presynaptic spike times, τ_*s*_ = 100 ms the decay time of NMDA currents, τ_*x*_ = 2 ms controls the rise time of NMDAR channels, and α_*s*_ = 0.5 kHz controls the saturation properties of NMDAR channels at high presynaptic firing frequencies. Parameters for synaptic transmission were taken from Compte et al. (2000).

**Figure 1:**
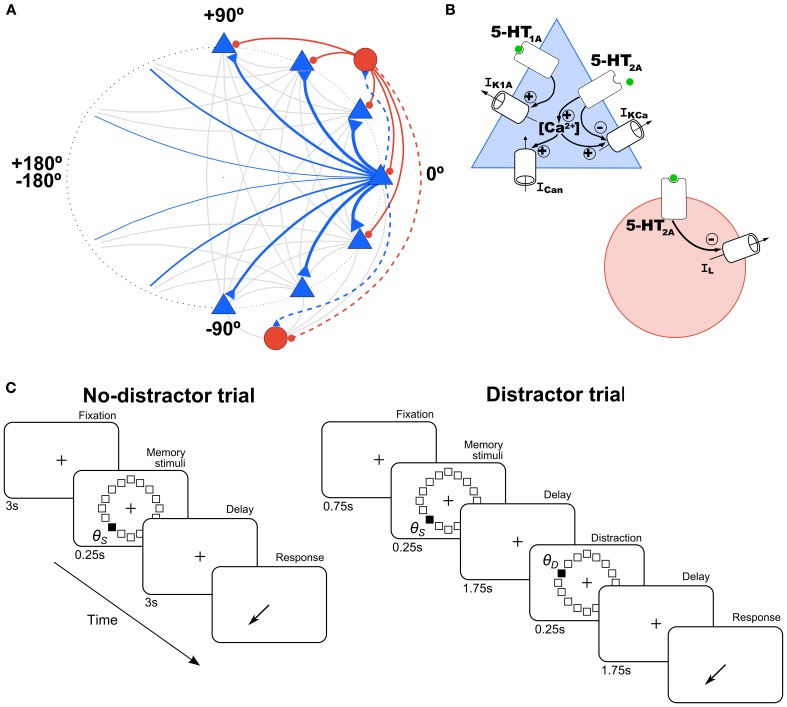
**Schematic of network architecture and 5-HT modulation in model neurons. (A)** Scheme of the ring structure of the network model with excitatory pyramidal neurons (blue triangles) and inhibitory interneurons (red circles) in a proportion of 4:1 interconnected within and between them. Nearby pyramidal neurons are strongly connected with each other (strength indicated by thickness of connections). Connections onto pyramidal neurons are indicated with a solid line and onto interneurons with a dashed line. **(B)** In pyramidal cells (blue triangle), 5-HT has an inhibitory effect via 5-HT_1A_ receptors by increasing a K^+^ current (*I*_*K1A*_), and an excitatory effect via 5-HT_2A_ receptors by inhibiting the Ca^2+^-dependent K^+^ current (*I*_*KCa*_), and increasing intracellular Ca^2+^, which modulates in turn a non-specific cationic current (*I*_*Can*_). In interneurons (red circle), 5-HT inhibits passive leak currents (*I*_*L*_) via 5-HT_2A_ receptors. **(C)** Schematic diagram of the simulated tasks. In the no-distractor trials (left), after 3 s of fixation a stimulus is presented in one of 16 possible locations. After a delay of 3 s subjects have to report the location of the stimulus. In the distractor trials, a distractor stimulus is presented at location θ_*D*_ during the delay (at 1.75 s), and a second delay of 1.75 s follows before the behavioral response.

The network model simulates neurons selective to the memorized location in working memory tasks (Funahashi et al., 1989; Goldman-Rakic, 1995). Pyramidal cells and interneurons were spatially distributed on a ring, labeled by their preferred direction of motion (θ_*i*_, from −180 to 180°) (Figure 1A). Connections between cells were spatially tuned, such that nearby cells were more strongly connected than distant cells (Compte et al., 2000). The connection strength *g*_syn, *ij*_ between pyramidal cells *i* and *j* depended on the difference in preferred angle between the cells and was described by the equation *g*_syn, *ij*_ = *W*(θ_*i*_ − θ_*j*_) *G*_syn_, where *W*(θ_*i*_ − θ_*j*_) was the sum of a constant term plus a Gaussian: *W*(θ_*i*_ − θ_*j*_) = *J*^−^ + (*J*^+^ − *J*^−^) exp[−(θ_*i*_ − θ_*j*_)^2^/2σ^2^]. *W*(θ_*i*_ − θ_*j*_) depends on two parameters, *J*^+^ and σ, while *J*^−^ is determined from a normalization condition (Compte et al., 2000). All the connections were structured with the same σ (σ_*EE*_ = σ_*EI*_ = σ_*IE*_ = σ_*II*_ = 14.4°) but with different *J*^+^: *J*^+^_*EE*_ = 2, *J*^+^_*EI*_ = 0.5, *J*^+^_*IE*_ = 1.4, *J*^+^_*II*_ = 1.9. Following the notations in Compte et al. (2000) and Cano-Colino et al. (2013), the parameters defining the strengths of local connections in the network were as follows: *G*_*EE, AMPA*_ = 0.14 nS, *G*_*EE, NMDA*_ = 2.1 nS (pyramid to pyramid); *G*_*EI, AMPA*_ = 0.72 nS, *G*_*EI, NMDA*_ = 1.9 nS (pyramid to interneuron); *G*_*IE*_ = 7.8 nS (interneuron to pyramid); *G*_*II*_ = 4.4 nS (interneuron to interneuron).

### 5-HT modulation

The model included 5-HT receptor physiological effects on PFC neurons (Figure 1B). 5-HT_1A_ and 5-HT_2A_ receptors are the two most abundant receptors in the PFC (Santana et al., 2004), which are highly co-localized in pyramidal neurons (~80%) (Amargós-Bosch et al., 2004). The main and faster inhibitory response to 5-HT pulses is through 5-HT_1A_ receptors and the later excitatory response through 5-HT_2A_ receptors (Puig et al., 2005; Goodfellow et al., 2009), which desensitize at high [5-HT] levels (Araneda and Andrade, 1991). We modeled the kinetics of the mechanisms triggered by these receptors with the following simple kinetic equations (Destexhe et al., 1994; Cano-Colino et al., 2013):

$$\begin{array}{l} \frac{ds_{1A}}{dt}=\frac{-s_{1A}}{\tau_{1A}}+\alpha_{1A}\text{​}\left[5\text{-HT​}\right]\text{​},\\ \frac{ds_{2A}}{dt}=\frac{-s_{2A}}{\tau_{2A}}+\alpha_{2A}[5\text{-HT}](1-s_{2A}) \end{array}$$

where *s*_1A_ and *s*_2A_ are the gating variables for the corresponding 5-HT receptors (0 < *s*_1A_, *s*_2A_ < 1); [5-HT] is the serotonin concentration, which we take to be 10 nM in physiological conditions (Celada et al., 2001), but the exact absolute value is not critical in our model. The receptor time constants τ_1A_ and τ_2A_ (τ_1A_ = 30 ms and τ_2A_ = 120 ms) were chosen to match the time course of PFC responses to the electrical stimulation of raphe neurons in the rat (Amargós-Bosch et al., 2004; Puig et al., 2005). The parameters α_1*A*_ = 1.8 kHz/μM, α_2*A, E*_ = 2.25 kHz/μM and α_2*A, I*_ = 11 kHz/μM control the affinity of the receptors to 5-HT and were obtained through an optimization procedure seeking to ensure the stability of working memory function in the network in the presence of baseline 5-HT (Cano-Colino et al., 2013). Assuming a diffuse, temporally constant action of 5-HT on the network's mechanisms to mimic systemic application of serotonergic agents, our simulations used a constant value of [5-HT] for all neurons in the network and through all periods of the task. Phasic actions of the serotonergic system in the course of the task are not evaluated in this study. In different simulations we changed the tonic level of [5-HT], increasing or decreasing the initial value (physiological level, see above) by 10 or 20%. We also mimicked the effect of agonists or antagonists of 5-HT receptors. To this end, we ran simulations in which we kept constant the activation of one receptor, by fixing the value of [5-HT] in its kinetic equation, while we modified the activation of the other one by changing [5-HT] in its equation.

The physiological actions of these receptors on pyramidal neurons have been characterized in *in vitro* electrophysiological studies (Andrade et al., 1986; Araneda and Andrade, 1991; Béïque et al., 2004; Villalobos et al., 2005; Zhang and Arsenault, 2005; Ma et al., 2007). In interneurons, 5-HT_2A_ receptors have been described in *in vitro* studies to increase neuronal excitability (Deng et al., 2007; Puig et al., 2010). We included these electrophysiologically defined effects of 5-HT receptors in our network model (Figure 1B, see below) (Meeter et al., 2006; Cano-Colino et al., 2013).

We simulated the hyperpolarizing action of the 5-HT_1A_ receptor in pyramidal prefrontal neurons by including a 5-HT-modulated K^+^ current (*I*_*K1A*_) in excitatory cells of our network (Figure 1B, blue triangle), modeled according to:

$$I_{K1A}=g_{K1A}s_{1A}\left(V-V_{K}\right)$$

where *g*_*K1A*_ = 29.7 nS is the maximal conductance of the channel and *V*_*K*_ = −70 mV is the potassium reversal potential. The depolarizing/excitatory response to 5-HT_2A_ receptor activation was simulated through 3 different mechanisms: increase in intracellular Ca^2+^, inhibition of Ca^2+^-activated afterhyperpolarization currents (*I*_*KCa*_), and activation of an afterdepolarization current mediated by a Ca^2+^-dependent non-selective cation channel (*I*_*Can*_). The calcium dynamics were modeled by the following equation:

$$\frac{d\left[{\text{Ca}}^{2+}\right]}{dt}=\alpha_{\text{Ca}}\sum_{sp}\delta \left(t-t_{sp}\right)-\frac{\left[{\text{Ca}}^{\text{2+}}\right]}{\tau_{\text{Ca}}}+\gamma_{\left[\text{5-HT}\right]}s_{2A}$$

where γ_[5-HT]_ = 0.41 nM/ms controls calcium flow through 5-HT_2A_ receptors, the [Ca^2+^] influx per spike is α_Ca_ = 0.1 μM and τ_Ca_ = 240 ms (Wang, 1998; Tegnér et al., 2002). These calcium dynamics affect *I*_*KCa*_ and *I*_*Can*_ according to:

$$\begin{array}{l} \;I_{KCa}=g_{KCa}\left(1-s_{2A}\right)\frac{\left[{\text{Ca}}^{2+}\right]}{\left[{\text{Ca}}^{\text{2+}}\right]+K_{D}}\left(V-V_{K}\right)\text{​},\\ \;I_{Can}=g_{Can}m_{Ca}^{2}h_{Ca}\left(V-V_{Can}\right),\\ \frac{dm_{Ca}}{dt}=\frac{m_{\infty }-m_{Ca}}{\tau_{Can}},\\ \;m_{\infty }=\frac{\alpha_{Can}\left[{\text{Ca}}^{2+}\right]}{\alpha_{Can}\left[{\text{Ca}}^{2+}\right]+\beta_{Can}},\\ \;\tau_{Can}=\frac{1}{\alpha_{Can}\left[{\text{Ca}}^{2+}\right]+\beta_{Can}},\\ \;h_{Ca}=\frac{1}{1+\exp\left(\left(\left[{\text{Ca}}^{2+}\right]-\beta_{h}\right)\text{​}/\alpha_{h}\right)} \end{array}$$

where *g*_*KCa*_ = 703 nS, *V*_*K*_ = −70 mV, *K*_*D*_ = 30 μM, *g*_*Can*_ = 36 nS, *V*_*Can*_ = −20 mV, α_*Can*_ = 0.0056 [ms·μM]^−1^, β_*Can*_ = 0.002 ms^−1^, α_*h*_ = 3 μM, β_*h*_ = 5 μM (Wang, 1998; Tegnér et al., 2002; Cano-Colino et al., 2013).

We included the depolarizing action of 5-HT_2A_ receptors on model interneurons (Figure 1B, red circle) by having 5-HT_2A_ receptors activation decrease the conductance of the leak current:

$$g_{L}=g_{L}^{*}\left(1-s_{2A}\right)$$

where g^*^_L_ = 26 nS.

### Simulations

We simulated a SWM task that resembled behavioral protocols used in monkey experiments (Funahashi et al., 1989; Williams et al., 2002). In brief, monkeys fixate on a central spot during a brief presentation of a peripheral cue and throughout a subsequent delay period. After this delay, they make a saccadic eye movement to where the cue had been presented in order to obtain a reward. To mimic this behavioral protocol in our simulations, simulation trials consisted of four periods: fixation (3 s), cue (0.25 s), delay (3 s), and response (Figure 1C, left). In the fixation period there were no additional external inputs to the network so it typically stayed in a spontaneous, unstructured firing state. In the cue period, a cue stimulus was applied at location θ_*s*_. This was simulated as current injection to each excitatory neuron in the network (labeled by θ_*i*_) of intensity *I*_*s*_(θ_*i*_) = *I*_1_ exp[μ_stim_(cos(θ_*i*_ − θ_*s*_) − 1)]. We used *I*_1_ = 0.235 nA and μ_stim_ = 10. During the delay, no stimulus was presented so that the network maintained the cue position in a stable pattern of network activation (activity bump).

The response period was not simulated explicitly, but a decoding algorithm was used to simulate behavioral responses during simulated tasks. The task consisted of reporting the location of the cue stimulus after the delay period within a predefined tolerance window. To obtain a behavioral response from our simulation trials we computed a population vector estimation (Georgopoulos et al., 1986; Lee et al., 1988) from the network activity at the end of the delay period. Thus, if {*n*_*i*_, *i* = 1 … *N*_*E*_} are the spike counts of all the excitatory neurons labeled by {θ_*i*_, *i* = 1 … *N*_*E*_} in a 50-ms window at the end of the delay period, the population vector was computed as the normalized sum of each neuron's selectivity vector *e*^*i*θ_*i*_^ (we use complex notation to operate with vectors in a compact manner) weighted by its spike count: *P* = (∑*n*_*i*_*e*^*i*θ_*i*_^) (∑*n*_*i*_)^−1^. We then extracted the modulus *C* and angle θ_*R*_ of the resultant population vector: *P* = *Ce*^*i*θ_*R*_^. For each individual simulation trial we took θ_*R*_ as the decoded location memorized in the network activity before response initiation, the *behavioral response*. Correct trials were those trials for which |θ_*R*_ − θ_*S*_| < 22.5°, where 22.5° is an arbitrarily defined window around θ_*S*_ to define correct trials.

In several simulations, we tested the influence of distractors on network performance. We define a distractor as an external stimulus of the same intensity and duration as the cue stimulus, but which appears after the cue and the nature of the task requires it to be ignored. The distraction trials consisted of: fixation (0.75 s), cue stimulus in the direction θ_*S*_ (0.25 s), delay 1 (1.75 s), distractor in the direction θ_*D*_ (0.25 s), delay 2 (1.75 s), and response (Figure 1C, right). Distractors were modeled as a cue stimulus (same strength, *I*_1_ = 0.235 nA and μ_stim_ = 10, same duration, 250 ms) but at a different location relative to the cue stimulus. We tested several distances between the stimulus position and the distractor position (θ_*D*_ − θ_*S*_, from 0 to 180° in steps of 11.25°) for different levels of baseline [5-HT] (see above). We ran 100 simulations for each condition ([5-HT] and distance θ_*D*_ − θ_*S*_), and then we computed the behavioral response (report location, θ_*R*_) at the end of the delay period, from which we had a measure of the distractability as θ_*R*_ − θ_*S*_ vs. θ_*D*_ − θ_*S*_.

### Numerical integration

The integration method used was a second-order Runge–Kutta algorithm with a time step of Δ*t* = 0.02 ms. The custom code for the simulations was written in C++.

## Results

We used a computational network model to investigate how 5-HT modulates WM function (Cano-Colino et al., 2013). This neuronal network model mimics the activity of neurons in PFC of monkeys performing a visuo-spatial WM task (Compte et al., 2000), and incorporates the cellular mechanisms of receptors 5-HT_1A_ and 5-HT_2A_ described in PFC neurons *in vitro* (Figure 1B) (Materials and Methods) (Cano-Colino et al., 2013). The network model consisted of 1024 pyramidal neurons (excitatory cells) and 256 interneurons (inhibitory cells), each coding for a stimulus location at a specific angle (Figure 1A). Any neuron connected to all other neurons in the network, and it received independent random inputs (assumed to be inputs from other brain regions). Connections between pyramidal cells coding for nearby stimuli were stronger than average (see Materials and Methods). With this general network organization, simulations produce neural activity consistent with PFC single-neuron data acquired during performance of visuo-spatial WM tasks (Funahashi et al., 1989; Compte et al., 2000).

We used this model to simulate behavioral tasks used in monkeys and humans to test SWM (Funahashi et al., 1989; Park and Holzman, 1992; Park et al., 1999). The task (Figure 1C, left) starts with a fixation period of 3 s followed by a brief presentation (0.25 s) of a cue stimulus (localized external current input to the network). The cue stimulus appeared in a random location restricted within a circle of given eccentricity from the fixation point. Thus, the cue stimulus location was entirely described by an angle value θ_*S*_ (−180° < θ_*S*_ < 180°). After cue stimulus extinction there was a delay period of 3 s, after which the location of the cue stimulus had to be reported based on the network's neural activity at the end of the delay period. We did not simulate explicitly the response period activity of the network (see Materials and Methods).

**Figure 2:**
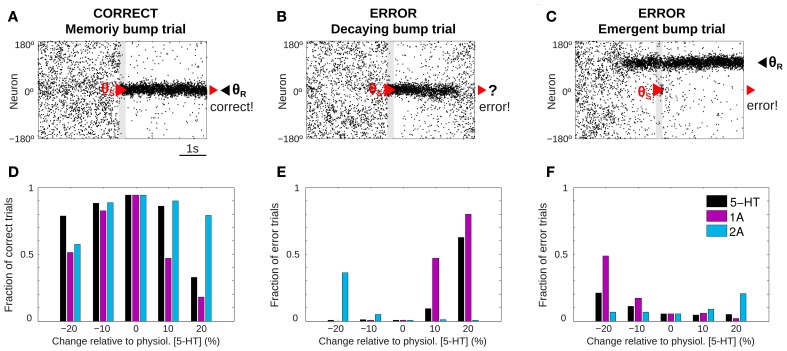
**5-HT modulates SWM performance in the network model. (A–C)** Example of rastergrams of simulations with 3 s fixation, a cue presentation of 250 ms (gray bar) and a delay period of 3 s. Panel **(A)** shows a correct trial: at the end of the delay period the memory bump is in the same position where the cue was presented. Possible errors can be due to: memory disappearance before the end of the delay period (**B**, decaying bump trial) or emergence of a spurious, misaligned bump (**C**, emergent bump trial). See specific criteria for defining correct and error trials in the text, based on the relative match of cue location (red triangle) and decoded bump location at the end of the delay (black triangle, θ_*R*_). **(D–F)** For different levels of [5-HT] (black bars), and varying activation of 5-HT_1A_ (purple bars) or 5-HT_2A_ (blue bars) receptors, fraction of correct trials **(D)**, fraction of decaying bump error trials, **(E)** and fraction of emergent bump error trials **(F)**. Thousand trial simulations per condition.

The network parameters could be tuned so that during the delay period the memory of the cue stimulus was maintained in a localized bump of neural activity (cluster of neighboring cells with raised activity, see Figure 2A) by virtue of strong reverberatory recurrent excitation among neighboring excitatory cells and strong disynaptic inhibition between excitatory cells of dissimilar selectivity (Compte et al., 2000; Tegnér et al., 2002). In this optimal network operation regime, this tuned persistent activity state (*memory state*) is maintained in a stable manner by the network, but the network can also sustain a low firing rate, unstructured network state (*spontaneous state*) if no cue stimulus has been presented (as during the fixation period in Figure 2A). Neuromodulation through 5-HT receptors imbalances this network regime and causes the destabilization of either the memory state or the spontaneous state, thus resulting in two types of behavioral errors that can be distinguished based on the reported confidence in the response (Cano-Colino et al., 2013). We first review briefly this result and we then use the model to derive additional predictions that do not require metacognitive evaluation.

### 5-HT modulation of SWM performance

We ran repeated trials with the task structure defined above but with different noise realization so that network activity over the course of the trial varied substantially from trial to trial. Then, we could extract behavioral output from these network simulations that could be treated similarly as in a real psychophysics experiment (Cano-Colino and Compte, 2012; Murray et al., 2012; Cano-Colino et al., 2013). For each simulation trial we obtained a *behavioral response* by extracting a population vector read-out of the angle θ_*R*_ encoded in network activity in a window of 50 ms at the end of the delay period. Thus, for each simulation trial we obtained the full network dynamics over the course of the trial, and one behavioral measure: the decoded stimulus location θ_*R*_. As it is usual in behavioral experiments, we classified trials as correct or error trials. If |θ_*R*_ − θ_*S*_| < 22.5° we classified the trials as correct, and if |θ_*R*_− θ_*S*_| > 22.5° the trial was an error. Typically, in correct trials the localized network activity (*bump*) triggered by the stimulus at θ_*S*_ was maintained by excitatory reverberation robustly through the delay (*memory bump trials*, Figure 2A). When inspecting the network dynamics in error trials two main causes for errors could be distinguished. In some cases, network activity formed in response to the stimulus at θ_*S*_ failed to reverberate through the length of the delay period so that by the end of the delay network activity was unstructured and did not contain any robust signal (*decaying bump trials*, Figure 2B). Responses in these error trials would be declared with low confidence because there was no signal in the network at the end of the delay and the response θ_*R*_ would essentially be randomly chosen. In other cases, the error occurred because a spontaneous bump of activity was formed in the network before the cue stimulus was presented and it remained stable for the duration of the delay period, despite the subsequent presentation of the stimulus at θ_*S*_ (*emergent bump trials*, Figure 2C). These responses would be declared with high confidence, as the late-delay signal in the network was strong, albeit wrong.

We then ran 1000 trials of this task simulation for 5 different values of tonic 5-HT concentration, and we identified trials as correct or error trials based on the classification described above. When the network model was subject to the reference 5-HT concentration of 10 nM (*physiological level*) almost all trials were correct responses. However, when the tonic 5-HT level was either increased or decreased, errors became more frequent (Figure 2D, black bars). The results showed an inverted U-shape, with optimal performance around our reference 5-HT concentration. Similar non-monotonic dependencies were observed when the activation of 5-HT_1A_ or 5-HT_2A_ receptors were independently manipulated (Figure 2D, purple and blue bars). It has been argued that this non-monotonic dependence of behavioral accuracy with 5-HT concentration could be a factor in the inconsistent results of psycho-pharmacological studies exploring the effect of 5-HT on SWM (Cano-Colino et al., 2013).

Inspection of the network activity in individual trials revealed that errors committed were very different in the high 5-HT and low 5-HT conditions: Errors in the low 5-HT network were mostly emergent bump trials (Figure 2F, black bars), while the performance decrease for high 5-HT concentrations was mostly due to decaying bump trials (Figure 2E, black bars). The same trend of error types was observed varying only 5-HT_1A_ receptor activation: 5-HT_1A_ agonists caused decaying bump trials (Figure 2E, purple bars) and 5-HT_1A_ antagonists promoted emergent bump trials (Figure 2F, purple bars). In contrast, 5-HT_2A_ receptor manipulations presented the opposite behavior: antagonists of 5-HT_2A_ increased decaying bump trials (Figure 2E, blue bars) and 5-HT_2A_ agonists increased the incidence of emergent bump trials (Figure 2F, blue bars). The analogy between the types of errors resulting from 5-HT_1A_ receptor activation and from 5-HT concentration increases, indicates that the relevant effect of 5-HT in this WM network is a change of cellular excitability in excitatory neurons (because this is the only effect of 5-HT_1A_ in the model) (Cano-Colino et al., 2013). One can therefore understand emergent bump trials as a consequence of enhanced network excitability, and decaying bump trials as a result of reduced network excitability. These two network dynamics cause behavioral errors of very different nature.

Non-monotonic dependencies of behavioral performance with 5-HT modulations in Figure 2D can thus turn into monotonic relationships (Figures 2E,F) if we can distinguish the nature of the errors (whether a decaying bump trial or an emergent bump trial) on a trial-by-trial basis. Monotonic dependencies would then be a lot easier to document in experimental population studies. In our network simulations we resorted to the full network dynamics, but this is experimentally inaccessible with current neurophysiological techniques. One possibility presented by (Cano-Colino et al., 2013) is to use the confidence in the response as a behavioral parameter to distinguish the two types of errors: low-confidence errors would mostly follow the predictions for decaying bump trials (Figure 2E), while high-confidence errors would mimic emergent bump trials (Figure 2F). We now turn into identifying other ways to tell apart the two error regimes experimentally without resorting to a metacognitive evaluation.

### Delay-length dependency of 5-HT effects on SWM performance

**Figure 3:**
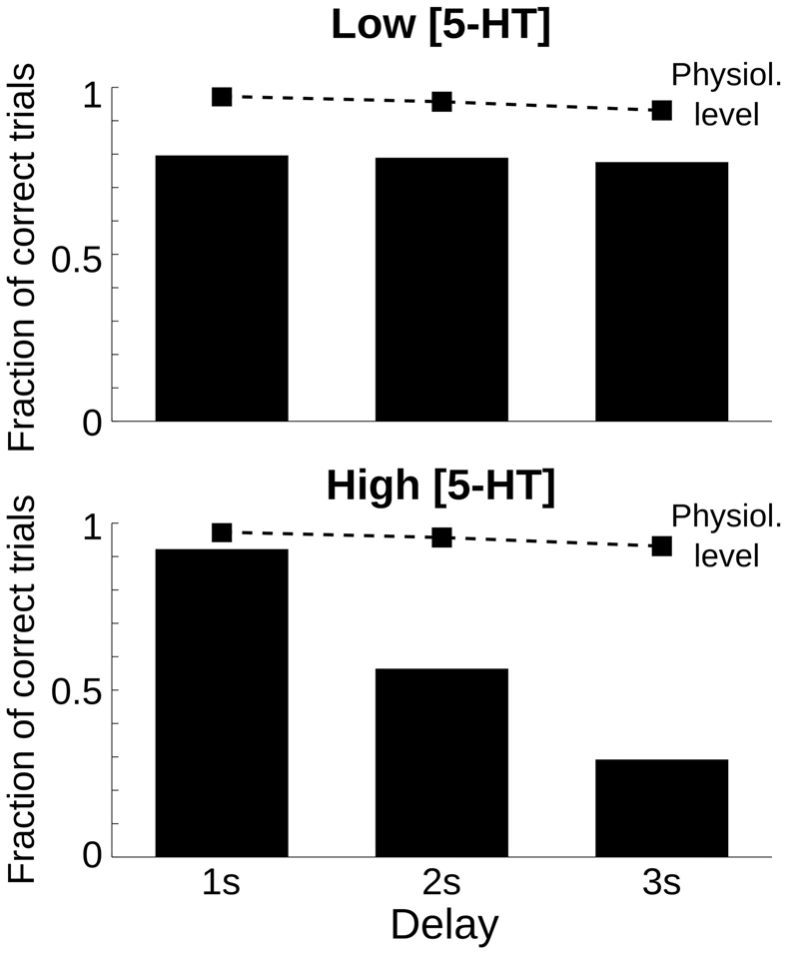
**Only high 5-HT effects on SWM performance depend on delay length**. Fraction of correct trials, from a total of 1000 simulation trials, for a delay length of 1, 2, and 3 s for two levels of [5-HT], compared with the physiological level of [5-HT] (dashed line). **Top**, for a low level of [5-HT] (20% reduction compared to the physiological level), the fraction of correct trials was reduced but remained roughly constant for different delay lengths. **Bottom**, for a high level of [5-HT] (20% increase compared to the physiological level), the fraction of correct trials diminished parametrically with increasing delay length.

One defining feature of working memory processes is the fact that the retention of stimulus attributes degrades with time (Pasternak and Greenlee, 2005). Also in our computational network model, the representation of the cue stimulus degrades during the delay (Compte et al., 2000). We reasoned that the performance of networks with parameter modulations promoting decaying bump trials would be especially sensitive to the duration of the delay because for longer delays more and more memory states would destabilize and decay to the spontaneous state. We tested this explicitly by running multiple trials with three different delay lengths: 1, 2, and 3 s delay, and for three 5-HT concentrations: physiological [5-HT], low [5-HT], and high [5-HT]. When we plotted the fraction of correct trials for each of the factors, we found that for increases in baseline 5-HT levels behavioral performance decayed with delay duration more strongly than in either the physiological or low [5-HT] conditions (Figure 3). Strong delay-length dependency of SWM performance therefore characterizes decaying bump error trials and should be a specific trait of agonists, not antagonists, of 5-HT_1A_ receptors or antagonists, not agonists, of 5-HT_2A_ receptors, or treatments leading to increased, not decreased, baseline [5-HT] according to our computational network model.

### 5-HT receptors effect on the distractibility of the network

The ability to resist distractors is an important component of SWM, and our computational network model has inhibitory mechanisms that allow it to resist the presentation of intervening distractors (Compte et al., 2000). We hypothesized that a regime with a fraction of decaying bump trials would behave radically different than a regime with a large proportion of emergent bump trials in relation to the filtering of unwanted distractors, and this would provide us with another prediction to disambiguate error types in different neuromodulatory manipulations. We therefore sought to characterize how 5-HT neuromodulation affected the resistance to distractors presented as intervening stimuli during the delay period in the network model.

**Figure 4:**
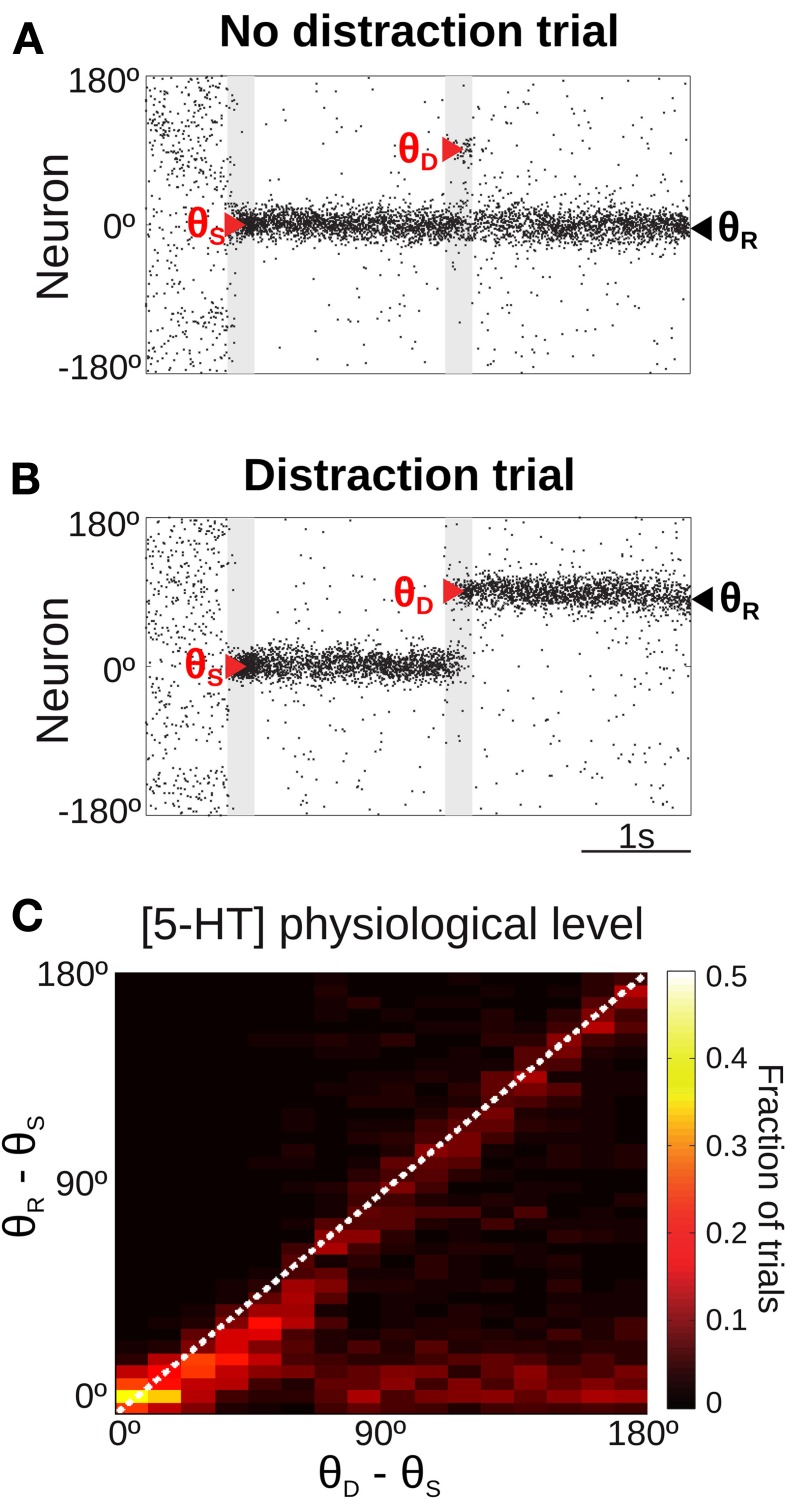
**Only for distant intervening distractors can the network model resist distraction. (A,B)** Rastergram during a simulated SWM task with a distractor. Transient cue presentation of 250 ms (first gray bar) centered at angle θ_*S*_ (first red triangle) induces a tuned sustained memory state, which could be maintained **(A)** or not **(B)** after the presentation of a distractor (second gray bar) of the same duration and intensity as the cue, but in a different position θ_*D*_ (second red triangle). The report location (θ_*R*_, black triangle) is decoded from activity at the end of the delay period. **(C)** For the physiological level of [5-HT], fraction of simulation trials in which a given distraction angle θ_*R*_ − θ_*S*_ was observed when stimulus and distractor were presented at different relative locations θ_*D*_ − θ_*S*_ (θ_*S*_ = 0°, θ_*D*_ from 0 to 180° in steps of 11.25°, 100 simulations per θ_*D*_). Warmer colors on the diagonal indicate distraction, while resistance to distractors is characterized by higher fraction of trials (warmer colors) along the *x*-axis.

To this aim, we ran trials with a different simulation protocol (distractor trials, Figure 1C, right) (see Materials and Methods). First, a cue stimulus was shown at an angle θ_*S*_. It triggered normally the memory state, a bump with a peak at an angle close to θ_*S*_ (see Figures 4A,B) prior to the presentation of a distractor at angle θ_*D*_, of the same intensity and duration as the cue stimulus (see Materials and Methods). We then quantified the effect of the distraction by measuring the peak location of the bump state at the end of the delay period (report angle θ_*R*_, Figures 4A,B) (see Materials and Methods). The effect of the distractor was quantified as the difference between the location of behavioral response compared with the location of the cue stimulus, θ_*R*_ − θ_*S*_. Distractor stimuli were presented at various positions relative to the cue stimulus (θ_*D*_ − θ_*S*_), in separate trials. The effect of the distractor depends on the stimulation intensity (Compte et al., 2000), so we considered only the condition in which the applied distractor stimulus had the same intensity and duration than the cue stimulus. We chose stimulation intensity so that a similar proportion of trials had response θ_*R*_ in a vicinity of the cue θ_*S*_ and the distractor θ_*D*_ stimuli, for large θ_*D*_−θ_*S*_.

When the distractor was applied, the network activity that was previously storing the cue stimulus could maintain its original position resisting the distraction (Figure 4A) or move to the location of the distractor (Figure 4B). We ran several simulations of the task for different distances between the stimulus position and the distractor position (θ_*D*_ − θ_*S*_). Figure 4C shows the fraction of trials that, for a given distance between the distractor and cue stimuli (θ_*D*_ − θ_*S*_), had an effect in the behavioral response θ_*R*_ − θ_*S*_ (distance between the location of the report and the cue stimuli). As previously shown (Compte et al., 2000), the effect of the distractor depended on the distance to the cue stimulus: distraction was very probable when distractor and cue stimulus where presented in nearby locations (θ_*D*_ − θ_*S*_ < 45°). When the distance θ_*D*_ − θ_*S*_ increased, both distraction and no-distraction trials were observed in a similar proportion (as a result of our choice of stimulation strength, see above). This pattern of distraction corresponded to our network model with a physiological level of [5-HT].

**Figure 5:**
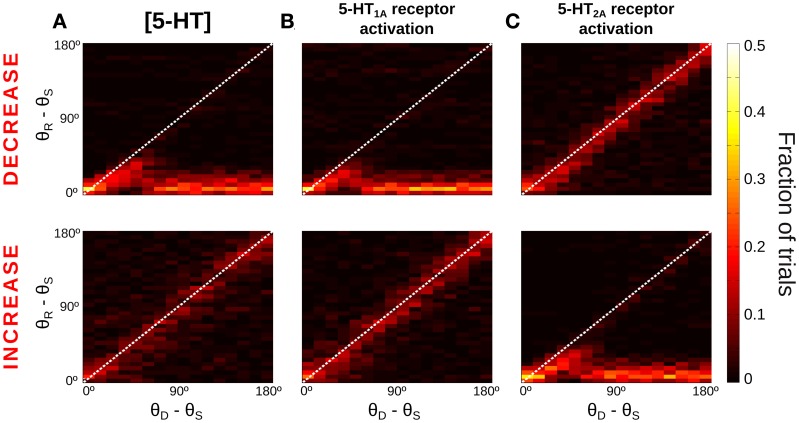
**More network distractibility for increasing [5-HT] levels**. Same as Figure 4C, for decreases (Top panels) or increases (Bottom panels) of [5-HT] and 5-HT receptor modulations. **(A)** Top, for low [5-HT] levels (−20% change relative to physiological value) the network shows persevering behavior, i.e., no distraction for any condition. Bottom, for high [5-HT] (+20% change relative to physiological value) the network always gets distracted. **(B)** 5-HT_1A_ antagonists (Top, −20% change relative to baseline 5-HT_1A_ activation) and 5-HT_1A_ agonists (Bottom, +10% change relative to baseline 5-HT_1A_ activation) have similar effects on network distractibility than corresponding manipulations of [5-HT], suggesting that general 5-HT modulation operates primarily on 5-HT_1A_ receptors. **(C)** In contrast, 5-HT_2A_ agonists (Top, +20% change relative to physiological value) and 5-HT_2A_ antagonists (Bottom, +20% change relative to physiological value) follow the opposite trend than corresponding [5-HT] modulations.

The manipulation of baseline 5-HT concentrations in the network model was found to greatly affect the distractibility of the network. Low [5-HT] (Figure 5A, top) led to perseverant behavior, resisting the distraction in almost all trials. Only when the distractor was within 45° of the cue stimulus the memory state was perturbed, and the memory bump was attracted toward the distractor location. For larger distances the network was unaffected by distractors. High [5-HT] (Figure 5A, bottom) had the opposite effect, the network resulted easily distracted by both close-by and distant distractors. Thus, the two conditions were more readily distinguished by using distractors sufficiently distant from the cue: if 33% of network simulations (293 out of 900) resulted in a correct response in the baseline condition for θ_*D*_ − θ_*S*_ > 90°, this fraction grew significantly to 76% of simulations when reducing baseline [5-HT] by 20%, and it instead decreased to just 6% of correct trials when baseline [5-HT] was raised by 20%.

We also studied how specific receptor agonists and antagonists would modify the capacity of the network to resist distractors. From the results of Figures 2E,F we hypothesized that agonists of 5-HT_1A_ receptors and antagonists of 5-HT_2A_ receptors would have the same effect on network distractibility as increasing baseline [5-HT], further supporting the idea that changes of [5-HT] engage dominantly 5-HT_1A_. Therefore, we varied in our network simulations the tonic activation of one receptor type independently from the other, mimicking the effect of agonists or antagonist of the receptors, similarly to what we did in Figure 2. When the activation of 5-HT_1A_ receptors was decreased (Figure 5B, top) the network was completely unaffected by distractors, as it happened for low levels of [5-HT] (Figure 5A, top). And when 5-HT_1A_ receptors were agonized the network lost its ability to resist the distractors for any position of the distractor (Figure 5B, bottom), as it also happened with high [5-HT] (Figure 5A, bottom). In contrast, modulation of 5-HT_2A_ receptors activation led to the reversed pattern: 5-HT_2A_ agonists made the network become more resistant to an intervening stimulus, whereas 5-HT_2A_ antagonists rendered the network very sensitive to intervening distractors (Figure 5C). The larger differences again occur for θ_*D*_ − θ_*S*_ > 90°, so that the percent of correct responses in these trials is 73% (5%) for a 20% 5-HT_1A_ (5-HT_2A_) receptor activity reduction, and 5% (74%) for a 20% 5-HT_1A_ (5-HT_2A_) receptor activity enhancement.

Thus, SWM tasks with intervening distractors is a behavioral protocol that can clearly tell apart the two different causes for SWM deficits that our network model predicts to occur as serotonergic neuromodulation is parametrically varied.

### 5-HT modulates the shape of neuronal memory fields

**Figure 6:**
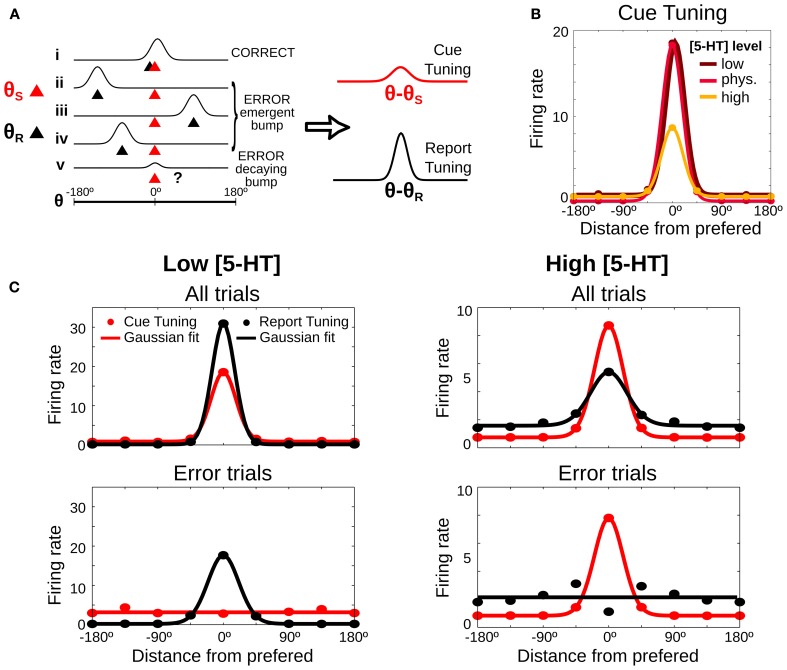
**Neuronal tuning curves change predictably depending on [5-HT] levels. (A)** Scheme of 5 possible examples of neuronal firing rate during the delay period (*i–v*). The red triangle shows the location of the stimulus (θ_*S*_), the black triangle the report location calculated at the end of the delay period (θ_*R*_) and the classification of each trial is showed on the right, computed as in Figure 2. Right, Tuning curves are computed by averaging firing rate, after aligning the trials to the stimulus location (red line, *cue tuning*) or to the report location (black line, *report tuning*). **(B)** Predicted delay cue tuning changes with 5-HT: low [5-HT] (dark red line) induces increases in tuning curve tails, high [5-HT] (orange line) mostly reduces tuning curve peak. **(C)** Tuning curves in the delay period computed from the stimulus cue position (red lines) and from the position of the behavioral report (black lines). Average delay firing rates for bins of 45° (circles) and Gaussian fit computed from all trials (200 trials) (top panels) or just from error trials (bottom panels). Left: low [5-HT] (20% decrease compared to the physiological level) has better tuning for report than cue tuning curves. Right: high [5-HT] (20% increase compared to the physiological level) shows better tuning for cue than for report tuning curves.

So far we have been able to propose two experimental validation of the computational model in behavioral experiments that do not require metacognition evaluation. However, our model is neurobiologically explicit and it can also produce predictions regarding PFC neural activity in SWM tasks upon systemic 5-HT neuromodulation. We derived one such prediction based on how the different error types affect neural tuning curves in the delay period of a SWM task. We first illustrate how we build such tuning curves schematically in Figure 6A, which shows schematically the firing rate during the delay period for 5 different trials (labeled *i–v*) in which the stimulus was presented at θ_*S*_ = 0 (red triangles), together with the location of the behavioral report (θ_*R*_, black triangles), computed at the end of the delay period. Following the criteria in Figure 2, trial *i* would be classified as a correct trial, trials *ii, iii*, and *iv* would be emergent bump error trials and trial *v* would be a decaying bump error trial. The latter shows a small bump around the cue location due to the activity at the beginning of the delay, before the bump decays, but the report location is in a random location, because the activity at the end of the delay is unstructured. As it is customary to build tuning curves, we can average the firing rate over many different trials by using the location of the cue stimulus as a reference (θ − θ_*S*_). We call this *cue tuning* (Figure 6A right, red curve). Error trials can have a marked influence on the cue tuning: although its height depends on correct trials, it decreases if there is a substantial number of error trials.

In addition, we now propose to compute a different neural tuning curve, based on the behavioral response. We calculate the *report tuning* curve (Figure 6A right, black curve) by averaging the firing rates during the delay period in different trials as a function of the distance from the neuronal cue preference θ (obtained from the cue tuning curve) to the report location θ_*R*_ (θ − θ_*R*_). The report tuning is also affected by the number of error trials, but it is in addition very sensitive to the type of error trials. Thus, emergent bump error trials (*ii–iv*) make the curve more sharply tuned, while decaying bump error trials (*v*) reduce its tuning. We therefore hypothesized that a systematic study of how serotonergic manipulations alter the sharpness of cue tuning and report tuning in a SWM task could yield a prediction from our modeling approach that could be tested electrophysiologically to identify the effects of different error substrates in PFC neuronal responses.

We used the full network dynamics from our simulations in Figure 2 to test this hypothesis with our modeling data. We computed neuronal firing rates in the delay period for each neuron θ in each simulation trial and we averaged across all trials, correct and error trials. We first analyzed predictions in relation to the classically defined delay tuning curve, the cue tuning. Relative to the cue tuning curve in the control [5-HT] levels, reduction of baseline [5-HT] essentially increased firing rate to non-preferred cues, while increased [5-HT] caused primarily a reduction of delay firing rates in response to cue stimuli (Figure 6B). We then computed delay firing rates using both bins centered around the cue location θ_*S*_ (cue tuning) and bins centered around the report location θ_*R*_ (report tuning). We found that for low baseline [5-HT] (Figure 6C, top left) the report tuning curve was more sharply tuned than the cue tuning curve because of the high incidence of emergent bump error trials (Figure 2). On the other hand, for high baseline [5-HT] (Figure 6C, top right) the report tuning curve presented worse selectivity than the cue tuning curve, as a result of decaying bump error trials. An increase in the firing rate in the tails of the curves was also observed for the high baseline [5-HT] condition, especially for the report tuning curve.

These differences were still bigger when we analyzed only error trials. For low baseline [5-HT] (Figure 6C, bottom left) the cue tuning curve showed essentially no selectivity for the cue because practically all errors corresponded to emergent bump trials. In contrast, the report tuning curve was still nicely tuned because these error trials maintained a bump of activity centered around the report location (Figure 2C). For high baseline [5-HT] (Figure 6C, bottom right) the cue tuning curve retained good cue selectivity because decaying bump error trials had bumps centered around the cue location in the early period of the delay. In contrast, the report tuning was not selective at all, since for these error trials responses do not reflect a bump of activity in the network but a random choice due to the lack of network selectivity at the end of the delay (Figure 2B). Note however a small selectivity in the report tuning of the bottom right panel in Figure 6C. This is due to the fact that mean network activity over the whole delay (as plotted in Figure 6C) still has residual enhanced rates around the cue stimulus for decaying bump trials (case *v* in Figure 6A), which are the dominant type of error trials there. Since in error trials behavioral reports are far from the cue, neurons with preference right on the report are never participating in such residual mean activity. This explains the dip in the report tuning curve in Figure 6C, bottom right panel.

Our computational model thus predicts that a comparison of cue tuning and report tuning curves from delay activity in SWM tasks should be able to reveal the different neural substrate of errors committed in the various 5-HT modulations considered. A majority of emergent bump errors leads to sharper report tuning than cue tuning, while a majority of decaying bump errors is reflected in a sharper cue tuning compared to the report tuning.

## Discussion

We used a cortical network model for SWM which includes the effect of 5-HT modulation of PFC neurons proposed previously (Cano-Colino et al., 2013) to investigate behavioral and physiological effects of 5-HT neuromodulation of SWM. The dynamics of our computational network model was strongly affected by changes in baseline 5-HT concentration [5-HT] in regard to the stability of the homogeneous, unstructured spontaneous state, and the stability of the tuned, persistent activity memory state (Cano-Colino et al., 2013). However, when the network was tested in a SWM task that mimics experimental behavioral protocols (Figure 1C, left), performance changed non-monotonically with [5-HT] (Figure 2D), which may compromise the detection of behavioral effects (Cano-Colino et al., 2013). However, the error types committed for reduced and increased [5-HT] were of radically different nature, either decaying bump errors (Figure 2B) or emergent bump errors (Figure 2C), and distinction between these error types revealed monotonous, predictable effects of [5-HT] manipulations that would be easier to prove experimentally (Figures 2E,F). In a previous study we argued that one way to distinguish error types would be by separating errors based on the declared confidence of the subjects in their responses (Cano-Colino et al., 2013). While confidence reports are increasingly being used in behavioral studies of SWM (Pessoa and Ungerleider, 2004; Middlebrooks and Sommer, 2011; Mayer and Park, 2012; Rademaker et al., 2012; Tanaka and Funahashi, 2012), interpretations suffer from our limited understanding of metacognition mechanisms. Here, we sought to explore other possible strategies to distinguish errors without using metacognition, in order to control for possible confounds and to provide simpler behavioral protocols that can be implemented more easily in animal studies. Two of these predictions concern behavioral experiments that can be carried out both on human and non-human subjects (Figures 3, 5), and one prediction is specific for electrophysiological experiments in monkey studies of SWM (Figure 6).

In a first prediction we propose to study the dependency of behavioral accuracy with delay-length as a way to disambiguate decaying bump errors from emergent bump errors. Decaying bump errors are very sensitive to delay length because once the cue stimulus triggers bump activity in the network, random fluctuations can destabilize the bump and they are more likely to occur the longer the delay period. Thus, delay-length dependency in error rates is a property of decaying bump error trials, and not of emergent bump error trials (Figure 3), and this could distinguish the behavioral errors committed for low and high [5-HT] experimentally without requesting a report of confidence from the subjects. For other neuromodulatory systems, pharmacological modulations can indeed have delay-dependent or delay-independent behavioral effects on working memory depending on dosage (Penetar and McDonough, 1983; Chudasama and Robbins, 2004).

The second prediction concerned the ability to resist distractors during the SWM task. Previous computational studies have studied the conditions for SWM distraction in similar neuronal network models (Compte et al., 2000; Brunel and Wang, 2001; Durstewitz and Seamans, 2002; Gruber et al., 2006; Murray et al., 2012). When the external distractor is presented close to the location of the cue stimulus, the memory report is invariably attracted toward the distractor location (Figure 4C), but when the distractor is beyond a given distance from the cue (in Figure 4 ~45°), one observes either a negligible effect of the distractor or a complete distraction (Figure 4) (Compte et al., 2000). The proportion of distraction trials for these distant-distractor conditions (Compte et al., 2000) as well as the window of short distances in which the distractor attracts the memory trace (Gruber et al., 2006; Murray et al., 2012) depend on the specific parameters of the network simulations and are therefore subject to possible neuromodulation (Compte et al., 2000; Brunel and Wang, 2001; Durstewitz and Seamans, 2002; Gruber et al., 2006). Interestingly, recent psychophysics studies have found evidence for this distance-dependent attraction of distractors in SWM (Stefan Van der Stigchel et al., 2007; Herwig et al., 2010), thus lending support to our network mechanism for the control of SWM distraction.

Our model makes testable experimental predictions about how 5-HT pharmacological manipulations affect distractibility in a SWM task with intervening distractors. Drugs that enhance brain 5-HT levels, such as selective serotonin reuptake inhibitors or tryptophan loading treatments, should decrease the ability to resist distractors (Figure 5A, bottom), and result in increased errors for distractors far away from cue stimuli. On the other hand, reducing 5-HT brain levels with tryptophan depletion should lead to better resistance to distant distractors (Figure 5A, top). Directly modulating 5-HT_1A_ and 5-HT_2A_ receptors should also affect distractibility: Antagonists of 5-HT_1A_ and agonists of 5-HT_2A_ receptors would facilitate resistance to distractors, while increased distractibility should be expected after treatment with agonists of 5-HT_1A_ and antagonists of 5-HT_2A_ receptors (Figure 5). Experimentally, we are not aware of any study that has investigated the effect of 5-HT drugs on distraction in such SWM tasks. Instead, the effect of 5-HT on cognitive flexibility is well-described (Robbins and Roberts, 2007). Several studies have shown a perseverative, inflexible behavior associated with prefrontal 5-HT depletion including a failure in error detection, altered responsiveness to punishment or loss of reward, and a deficit in inhibitory control (Deakin, 1991; Murphy et al., 2002; Evers et al., 2005). Large depletions of 5-HT throughout the PFC (Clarke et al., 2004, 2005) as well as more restricted lesions targeting the OFC (Clarke et al., 2007) result in impaired discrimination reversal performance characterized by marked response perseveration. Although these tasks are clearly different from the distraction SWM task that we analyzed here, it is suggestive to realize that if we change the task and ask the network to switch to the memory of the distractor to complete the task, reduced [5-HT] would result in perseverant behaviors (memories would remain on the initial cue) and the network would be unable to perform the required switch.

We observed a parallelism in distractibility between the global effects of baseline [5-HT] and the activation of 5-HT_1A_ receptors. In contrast, distractibility effects from the activation of 5-HT_2A_ receptors followed the opposite trends, with agonists reducing distractibility and antagonists increasing it. These results reinforce the idea advanced before (Figure 2 and Cano-Colino et al., 2013) that 5-HT_1A_ receptors drive the effects of general modulation of 5-HT baseline levels in PFC in SWM.

As a third prediction, we took advantage from the fact that our model is mechanistically explicit to derive predictions at the neurophysiological level that can be addressed in electrophysiological experiments in behaving animals. We reasoned before that microinfusion of serotonin receptor agonists and antagonists in the PFC should alter behavioral WM performance non-monotonically (Figure 2), while modulating monotonically the average firing rate during delay periods of correct trials (Cano-Colino et al., 2013). We now add that in such experiments, receptor modulations should alter the shape of neuronal tuning curves in error trials (Figure 6). In error trials, delay tuning to the cued stimulus (cue tuning) should be diminished relative to correct trials but some residual cue tuning may be detectable, since decaying bump error trials have early-delay rate elevations linked to the cued location (Figure 2B). We propose to compare this cue tuning to tuning curves computed based on the behavioral report (report tuning), not the memory cue. Report tuning in the delay will be stronger (weaker) than cue tuning for emergent bump (decaying bump) error trials (Figure 6). Considering the specific effects of the 5-HT receptors separately, our model thus predicts stronger (weaker) report tuning than cue tuning in error trials after microinfusion of a 5-HT_1A_ antagonist (agonist) or a 5-HT_2A_ agonist (antagonist) (Figure 6). Although this difference is especially notorious for error trials, a change in tuning sharpness of cue and report tuning curves should also be detectable when analyzing all trials together, provided the task was designed to be near psychophysical thresholds so errors would be numerous.

Our study has some limitations. We included only 5-HT receptor mechanisms that are known to be in PFC neurons and have also been described physiologically in *in vitro* studies. We therefore did not include 5-HT_1A_ receptors on inhibitory interneurons or the effects of 5-HT_1A_ and 5-HT_2A_ receptors on intracortical glutamatergic and GABAergic synaptic transmission in our model (Celada et al., 2013). We also left out other receptors that are known to be expressed in PFC but lack *in vitro* characterization, such as 5-HT_3_ (Puig et al., 2004), or 5-HT_2C_ receptors (Pompeiano et al., 1994). However, analysis of the network simulations leads to the conclusion that the effects reported here are primarily dependent on the modulation of the pyramidal neuron excitability and not specific of the mechanisms of one single receptor (Cano-Colino et al., 2013). For this reason, we expect that adding more 5-HT receptors or functional effects as their *in vitro* characterization becomes available will increase the predictions that emanate from the model but will not essentially change the current findings. A second limitation is related with the fact that we are modeling neurons as single compartments where different 5-HT receptors interact compactly. However, there are indications of a possible distinct spatial distribution of 5-HT_1A_ and 5HT_2A_ receptors on PFC pyramidal neurons (Nichols and Nichols, 2008; Celada et al., 2013). Our modeling study cannot address possible mechanistic consequences of such segregation of 5-HT receptors on different neuronal compartments. A third limitation stems from the fact that our simulations rely on many parameters that lack clear experimental references. We reported here simulations that correspond to one particular network realization that yields reasonable SWM behavior, and the question remains as to how general these findings are in relation to other possible parameter instantiations. This is a difficult problem that affects this kind of modeling projects, but we have addressed it partially by finding 20 different network realizations using an unbiased automated optimization procedure and confirming that our predictions are shared by all these different networks (Cano-Colino et al., 2013). We are therefore confident that our analysis extends to a large family of SWM models with 5-HT neuromodulation.

Although we focused our study on 5-HT effects on SWM, the modulations of network function that we observed depend essentially on a concerted change in neuronal polarization in our network, here due to the action through 5-HT_1A_ receptors (Cano-Colino et al., 2013). Therefore, any neuromodulatory agent that alters the excitability of neurons in the PFC would lead to analogous predictions in our SWM network model. This is further strengthened by the fact that synaptic strengths are also known to affect emergent and decaying bump dynamics in a similar way to cellular excitability (Edin et al., 2009; Wei et al., 2012), so the qualitative results would apply to neuromodulators affecting either cellular or synaptic excitability. Thus, all our predictions aimed at distinguishing error types to characterize the behavioral effects of neuromodulation on SWM would apply also to the dopamine or norepinephrine (NE) systems (Aston-Jones and Cohen, 2005; Cools and D'Esposito, 2011). This is underscored by comparing qualitatively our results with those of Eckhoff et al. (2009), who also used a computational approach to explain the non-monotonic effects of NE on decision-making. They found that low tonic NE produces unmotivated behavior, due to fading or decaying memory. In contrast, high tonic NE causes impulsive responses and poor accuracy, due to the emergence of spontaneous activity prior to stimulus onset. Our results with 5-HT suggest that a careful analysis of error types in a wide range of cognitive tasks is critical to understand the effects of neuromodulatory systems in cognitive function. Experimentally, rodent experiments with D1 receptor manipulations report that an excessive activation leads to perseverative responses while suppression of the receptor induces more random behavior in a SWM task (Zahrt et al., 1997; Floresco and Phillips, 2001; Seamans and Yang, 2004). This is reminiscent of our findings that behavioral inverted U-curves may reflect different types of errors, and it is in line with the pattern of distractibility of the network upon modulation of 5-HT_2A_ receptors. Notably, D1 and 5-HT_2A_ receptor activations have both a generally depolarizing effect on PFC pyramidal neurons (Araneda and Andrade, 1991; Yang and Seamans, 1996). In a suggestive study (Vijayraghavan et al., 2007), local activation of D1 receptors by iontophoresis was associated with a dose-dependent change in neuronal tuning curves in a SWM task so that insufficient D1 stimulation led to diminished cue tuning due to stronger response to non-preferred locations, while excessive D1 stimulation diminished cue tuning by reducing responses to preferred locations. Interestingly, these modulations parallel our network model's predictions for 5-HT_2A_ receptor modulation (Figure 6B). Taken together, we propose that identifying error mechanisms when neuromodulation causes inverted-U dose-response curves in working memory can be a fruitful avenue to advance our understanding of neuromodulatory control of higher cognitive functions.

### Conflict of interest statement

The authors declare that the research was conducted in the absence of any commercial or financial relationships that could be construed as a potential conflict of interest.
